# Three-dimensional organization and dynamics of the genome

**DOI:** 10.1007/s10565-018-9428-y

**Published:** 2018-03-22

**Authors:** Przemyslaw Szalaj, Dariusz Plewczynski

**Affiliations:** 1 Centre for Innovative Research, Medical University of Bialystok, Białystok, Poland; 2 I-BioStat, Hasselt University, Hasselt, Belgium; 3 Centre of New Technologies, University of Warsaw, Warsaw, Poland; 4 Faculty of Mathematics and Information Science, Warsaw University of Technology, Warsaw, Poland

**Keywords:** Genome organization, Cohesin, CTCF, Chromatin loops, Topological domains, Compartments, Chromosome territories

## Abstract

Genome is a complex hierarchical structure, and its spatial organization plays an important role in its function. Chromatin loops and topological domains form the basic structural units of this multiscale organization and are essential to orchestrate complex regulatory networks and transcription mechanisms. They also form higher-order structures such as chromosomal compartments and chromosome territories. Each level of this intrinsic architecture is governed by principles and mechanisms that we only start to understand. In this review, we summarize the current view of the genome architecture on the scales ranging from chromatin loops to the whole genome. We describe cell-to-cell variability, links between genome reorganization and various genomic processes, such as chromosome X inactivation and cell differentiation, and the interplay between different experimental techniques.

## Introduction

One of the greatest milestones of the modern biology was the discovery of the DNA double helix structure by Watson and Crick in 1953. This discovery explained how the genomic material can be replicated and passed to further generations and laid the groundwork for future genome research. In the 1970s, as the electron microscopy techniques had matured, they eventually became capable of capturing chromatin fibers and nucleosomes. Another microscopy-based technique, fluorescence in situ hybridization (FISH) was instrumental in many discoveries related to genome organization (Langer-Safer et al. 1982). The discovery of nuclear ligation assay, a method that allows to determine the circularization frequencies in DNA (Cullen et al. 1993), inspired chromosome conformation capture (3C) (Dekker 2002) technology and gave rise to a multitude of 3C-based techniques such as 5C (Dostie and Dekker 2007) or ChIA-PET (Fullwood et al. 2009). Of particular interest is Hi-C, a high-throughput technique for capturing genome-wide chromatin interactions (Lieberman-Aiden et al. 2009) that led to many discoveries and became a de facto standard in the field. An extensive review of these techniques and the roles they played in shaping our current understanding of the genome topology can be found in Fraser et al. (2015b).

In this review, we describe the hierarchy of spatial genome organization on the scales ranging from chromatin loops to whole chromosomes. A special emphasis is put on the interplay between genome architecture and its function and on the functional role of the genome reorganization at different hierarchy levels.

## Genome organization

**Fig. 1:**
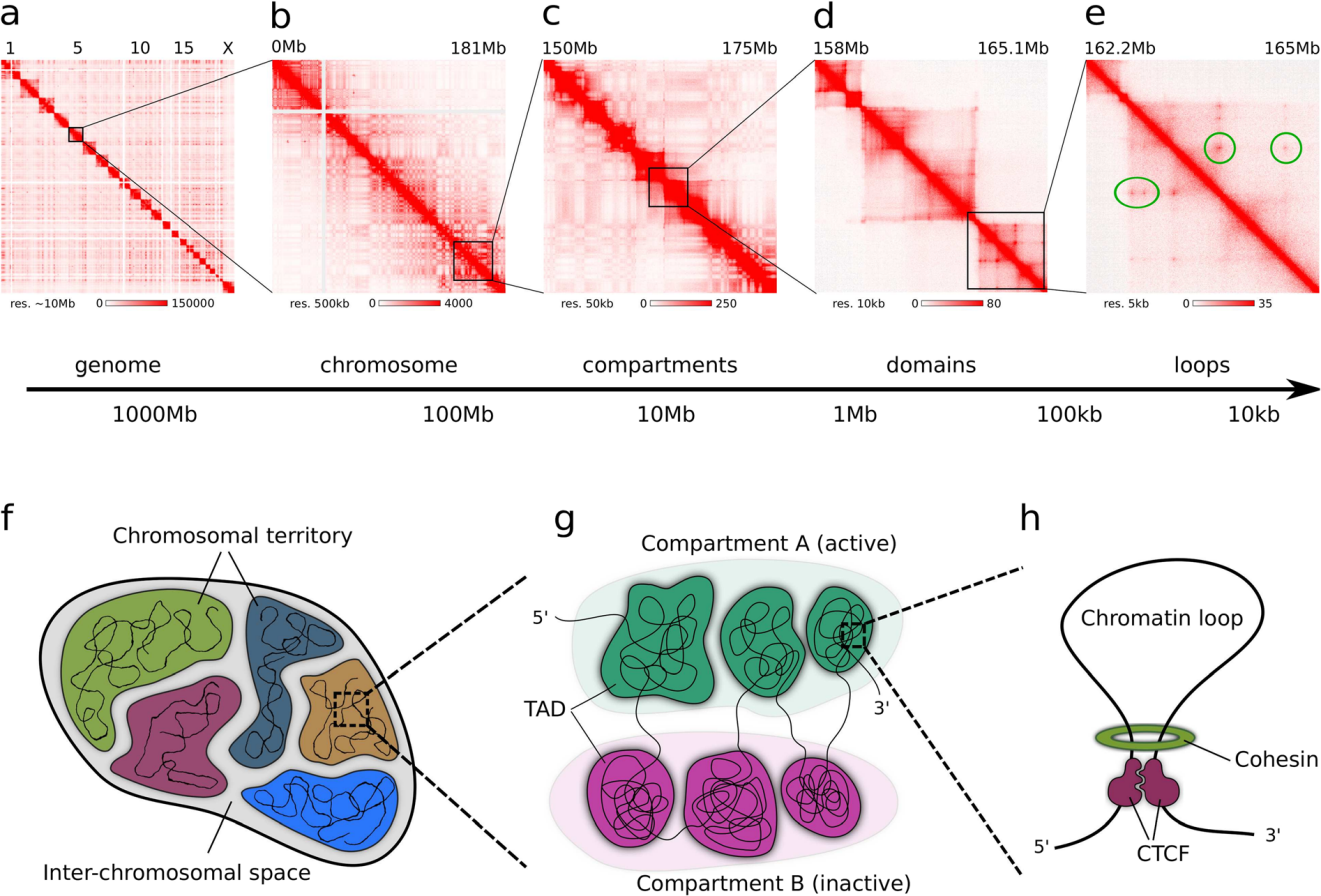
Hierarchical genome organization. Hi-C heatmaps for different scales: whole genome (**a**), whole chromosome (**b**), megabase (**c**, **d**) and hundred kilobases (**e**), and a model of genome folding at these scales (**f**–**h**) is shown. Whole-genome contact maps show that chromosomes occupy separate chromosomal territories and rarely interact with each other (**a**, **f**). Megabase-level heatmaps with clear square formations along the diagonal are indicative of topological domains (**c**, **d**, **g**). Plaid-like pattern corresponding to compartments A and B is also visible (**b**, **c**, **g**). Individual peaks corresponding to chromatin loops are clearly seen on the high-resolution heatmaps (**e**, **h**). Heatmaps were created from the GM12787 in situ Hi-C dataset published by Rao et al. (2014) using Juicebox (Durand et al. 2016)

The human genome is constituted of chromatin—a mixture of DNA, histones, and other DNA-binding proteins. DNA is wrapped around histones forming a flexible 10-nm fiber, which for a long time was believed to organize into a stiffer 30-nm array. However, a number of studies have questioned the existence of the 30-nm fiber in vivo, suggesting that even on this scale, chromatin is much more flexible and dynamic than previously thought (Nishino et al. 2012; Ricci et al. 2015; Ou et al. 2017; Maeshima et al. 2010). While it seems that there are no well-defined higher-order structures, chromatin takes during interphase genome topology is far from random, with multiple organizational units and principles governing its folding (Fig. 1).

### Chromatin loops

Chromatin is able to form long-range interactions in which two distant DNA segments are brought close to each other forming a loop (Fig. 1h). These loops exhibit a great variability in their length (with a range from a few kb to more than 100 Mb) and duration time (with both short, temporal loops created dynamically and strong, persisting loops existing for a significant part of the cell cycle), and they may link loci located both on the same or on different chromosomes. Recent studies suggested that different types of loops may be involved in various cellular mechanisms. One of such well-studied function of the loops is to bring together distant enhancers and promoters (Kadauke and Blobel 2009; Bulger and Groudine 2011). Enhancer-promoter interactions and other types of loops will be described in the following subsections.

It is difficult to determine the exact number of chromatin loops in a genome of an organism. Rao et al. detected approximately 10,000 loops in 5 kb Hi-C heatmaps in the GM12878 cell line (Rao et al. 2014) and Tang et al. identified over 42,000 CTCF and cohesin-mediated interactions using ChIA-PET (Tang et al. 2015); yet, some studies suggested the existence of as many as 100,000 (Sanyal et al. 2012), 175,000 (Javierre et al. 2016), and even 1,000,000 (Jin et al. 2013) loops.

The vast majority of the loops are short-ranged and operate locally, often within the boundaries of a topological domain, with a span less than 2 Mb (Rao et al. 2014; Tang et al. 2015), but there are also long-range interactions extending over tens of megabases or between different chromosomes. For example, chromosome X inactivation is associated with long-range chromatin loops between DXZ4, FIRRE, and G6PD loci (Tang et al. 2015).

### Enhancer-promoter

Deng et al. showed that gene transcription can be induced by targeted tethering between an enhancer and promoter, even when the key transcriptional activator is absent (W. Deng et al. 2012). On the other hand, the enhancer-promoter looping may not be sufficient for gene activation. In one study, it was discovered that while *zone of polarizing activity regulatory sequence* (ZSR) colocalizes with the *Sonic hedgehog* (Shh) gene in developing mouse limb bud cells, the expression only occurs in posterior cells, in which *Shh* loops out from the chromosomal territory (Amano et al. 2009). Enhancer-promoter interactions are closely related to transcriptional activity: they are generally established concomitantly with gene expression and disrupted when the genes become repressed (Bonev et al. 2017). This is, however, not a rule, as exemplified by the HoxD gene cluster, in which these interactions are present even in tissues where the corresponding genes are not expressed (Ghavi-Helm et al. 2014).

In a recent study, a YY1, a protein that was previously linked to many functions, such as activation, repression, differentiation, or cellular proliferation, was demonstrated to significantly contribute to enhancer-promoter interactions. It was shown that depletion of YY1 or deletion of its DNA binding sites can disrupt enhancer-promoter interactions and impact gene expression. In a study, YY1 depletion resulted in significant changes in expression of ~ 8000 genes, with half of them being upregulated, and half of them downregulated (Weintraub et al. 2017).

Early models often assumed that enhancers interacted with the nearest gene promoters, but presently, it is known that this looping often bypasses a number of enhancers between a gene and its regulatory element (Carter et al. 2002; Jeong 2006; Pennacchio et al. 2006; Ruf et al. 2011; Sanyal et al. 2012). For example, in embryonic stem cells (ESCs), 66% of the active promoters contact the nearest enhancer and 30% bypass at least one of them (the remaining 4% contact the enhancer that is closest up- or downstream, whereas a closer enhancer is present in the opposite direction). Interestingly, even in these 66% when promoter interacts with the closest enhancer, 90% of them interacts with at least one more distant enhancer (Schoenfelder et al. 2015a).

This looping is not limited to simple one-to-one associations, and in fact, it often forms complex multi-way interaction networks, where a single gene can be regulated by multiple enhancers, enhancers may have more than one target gene, and co-regulating genes may interact with each other (Gheldof et al. 2010; Tolhuis et al. 2002; Schoenfelder et al. 2009; Apostolou et al. 2013; de Wit et al. 2013; Denholtz et al. 2013; Ong and Corces 2011).

One study found that in mouse embryonic stem cells (mESC), 25% of promoters did not interact with any enhancer, 40% interacted with several (2–10), and 12% with more than 10 enhancers, with the level of expression being correlated with the number of enhancers. Seventy percent of enhancer-like elements were contacted by 1–5 promoters and 2–4% by more than 5 promoters. Additionally, super-enhancers do not enter contacts with more promoters than highly connected enhancers, but they tend to regulate highly expressed genes (Schoenfelder et al. 2015a).

These results are consistent with a more recent study in which promoter capture Hi-C was used to identify interacting regions in 17 human primary hematopoietic cell types (Javierre et al. 2016). In this study, a total of 175,000 interactions between promoters and promoter interacting regions (PIR) was detected, with a median of four interactions per promoter. Conversely, more than a half of PIRs interacted with only one promoter, and fewer than 10% interacted with four or more, suggesting that gene expression can be regulated by multiple regulatory elements. Approximately 10% of these contacts happened between loci more than 1 Mb apart, and ~ 5000 connected loci located on different chromosomes.

A slightly different view on these interactions was taken in another study, where all the regions interacting with a particular promoter were analyzed collectively as a single unit, termed a *cis-regulatory unit* (CRU) (Freire-Pritchett et al. 2017). The authors found ~ 9000 CRUs in human embryonic stem cells (ESCs) and ESC-derived neuroectodermal cells (NECs). The CRU lengths varied greatly from 1 kb up to 200 Mb, with a median of ~ 230 kb. In both cell types, 75% of CRUs were fully contained within TADs, implying that some of the interactions crossed TAD boundaries. Interestingly, while the boundaries that were crossed were generally weaker than non-crossed ones, the difference was not large, and even very strong boundaries were occasionally crossed.

An analysis of chromatin states showed that in 50% of CRUs, all PIRs exhibited one predominant chromatin state (either active, repressed, or poised), compared to ~ 20% of CRUs in which PIRs had mixed states (PIRs in the remaining CRUs were in the background state). Interestingly, in general, the prevalent CRU chromatin state determined the chromatin state of a corresponding promoter.

It was shown that enhancers generally have an additive effect on the expression of the target genes (Schoenfelder et al. 2015a; Javierre et al. 2016). This may be seen as an evolutionary security measure—as the regulatory role is distributed between multiple enhancers, the potentially pathogenic effect of a mutation in a single enhancer is limited. Additionally, the dynamic nature of these networks makes it possible that an ad hoc rewiring may happen to mitigate the effects (Javierre et al. 2016). These mechanisms may explain why many SNPs in non-coding regions are not detected as eQTLs even though they are located at the regulatory regions (Guo et al. 2015a). Yet, in some cases, even a disruption of a single enhancer may lead to serious phenotypic consequences. For example, one study showed that the experimental deletion of a limb-specific Shh enhancer leads to complete loss of Shh expression and subsequent limb degeneration (Sagai 2005).

The above results show that enhancer-promoter interactions form a variety of complex, highly dynamic, and cell-type specific networks. Whether there is some hierarchy of enhancers, with some of them being more important than the others, and how to predict the impact of a particular enhancer on its target gene expression still remain open questions. The situation is even more complicated by the fact that some of the PIRs lack typical enhancer-like features, but they still have an additive effect on gene expression. It is not yet known whether this effect is due to regulatory, structural, or topological roles, or if it is just an artifact of past interactions (Javierre et al. 2016). It is also not known whether multiple enhancers targeting a single gene are orchestrated to work simultaneously or sequentially, or if different sets of enhancers are involved in the transcription at different times or in different cell lines, providing an additional layer of selective gene regulation.

### Promoter networks

Another type of loops was observed between gene promoters. These interactions result from preferential contacts of various transcription factors (TFs) with their associated genes, which lead to establishment of gene clusters. Such clusters were observed for example for Klf1 in mouse fetal liver cells (FLCs) or Klf4 and pluripotency factors such as Oct4, Sox2, and Nanog in mESC (Schoenfelder et al. 2009; Wei et al. 2013; Apostolou et al. 2013; Denholtz et al. 2013). This allows a formation of specialized nuclear hot spots that share resources and allow for efficient transcription (Schoenfelder et al. 2009). But it seems that there is higher degree of coordination between genes in a cluster than just preferential colocalization. In one study, it was shown that the experimental removal of a single gene from such a complex directly affected the transcription of co-associated genes (Fanucchi et al. 2013).

Genome-wide analysis of such networks in mESC, neural progenitors (NPCs), and cortical neurons (CNs) cells has shown that, as opposed to enhancer-promoter loops, these interactions are not mediated by CTCF and that they often span across domain boundaries, suggesting that different mechanisms are responsible for formation of these two types of loops (Bonev et al. 2017).

Interestingly, the promoters involved in these interactions form functional networks which change during differentiation. For example, the strongest subnetwork in mESC (containing development-related genes) was absent in FLC, and in its place, the cell cycle and DNA replication-related subnetworks appeared (Schoenfelder et al. 2015a).

While these networks consist of active genes exclusively, it was found that genes generally tend to colocalize with other genes with a similar expression level, even for genes with medium and low expression (Schoenfelder et al. 2015a). It is difficult to explain this phenomena. While sharing the transcription sites explains colocalization of highly active genes, it is not known what mechanism could be responsible for colocalization of poorly expressed ones. It is possible that these results are due to a technical issue, as the specificity of the method used (promoter-capture Hi-C) makes it impossible to assess the enrichment of the interactions relative to the background. Yet, the successful identification of the active-gene networks suggests that the method in fact captures meaningless interactions. Moreover, the associations between promoters bound by key transcriptional regulators were higher than expected and could not be explained by the expression level and genomic distance, suggesting that there are some non-transcription-related factors involved in these contacts (Schoenfelder et al. 2015a).

Taken together, these results suggest the existence of a cell-type-specific dynamic system of interconnected genes and TF networks which play an important role in the gene regulation. This complex mechanism may be based not only on the spatial clustering of genes and regulatory factors that increase the effectiveness of transcription machinery, but also on the cooperation between all these elements.

### Gene loops

Another type of loops, gene loops, exist between the gene promoter and its transcription termination site (Bonev and Cavalli 2016; O’Sullivan et al. 2004). It was suggested that these loops may play several roles, such as enforcing a transcription directionality on bidirectional promoters, or facilitate recycling of polymerase (Tan-Wong et al. 2012) and transcription memory, a mechanism that allows genes to be rapidly transcribed (Lainé et al. 2009; Tan-Wong et al. 2009). While this looping is not required for transcription to start, it promotes subsequent gene reactivation, suggesting that some transcription products persist and affect its future regulation (Singh and Hampsey 2007; Tan-Wong et al. 2009). This effect is epigenetic and can be inherited, but it is not yet known how this information is retained (Brickner et al. 2007; Kundu et al. 2007).

In a recent study, aggregated single-cell heatmaps revealed that promoters of the highly active genes interact with the whole gene body, rather than just with the termination site. Interestingly, it was found that these contacts are strongly correlated with the number of gene exons, suggesting that they may be involved in splicing (Bonev et al. 2017)).

### Polycomb interactions

Another important group of interactions is polycomb-mediated looping. Polycomb group proteins play an important role in cell development both by complete silencing and dynamic regulation of genes required by developmental changes (Delest et al. 2012), as studied in Drosophila (Bantignies et al. 2011) and mammalian cells (Denholtz et al. 2013; Vieux-Rochas et al. 2015; Schoenfelder et al. 2015b).

A recent study showed that interactions between polycomb bound promoters form multi-way interaction networks capable of regulating genome architecture by silencing lineage-specific developmental genes. Of particular interest, there was a strong cluster of 4 Hox gene clusters, associated with 66 other genes (located on different chromosomes), mostly related to early developmental transcription factors linked to body plan specification, morphogenesis, and organogenesis regulation. Polycomb removal led to widespread enhancer activation and upregulation of previously repressed genes, based on which the authors hypothesized that the selective release of genes from this silencing network may be an important mechanism of cell fate specification (Schoenfelder et al. 2015b).

The precise mechanism of polycomb-mediated repression is not yet well understood. In particular, it is not known what is the relative importance of PRC1 and PRC2, two major types of polycomb repressive complexes. An earlier work implicated EED (a PRC2 component) to be required for establishment and/or maintenance of these long-range interactions. While in the EED absence, the contacting loci remained spatially collocalized, the frequency of interactions significantly decreased (Denholtz et al. 2013). However, it was also shown that PRC1 knock-out also significantly disrupts these contacts (Schoenfelder et al. 2015b), suggesting that both PRC1 and PRC2 are crucial to maintain these networks.

### Summary

Recent discoveries paint a picture in which DNA looping form complex networks of interactions between multiple loci. While the enhancer-promoter interactions are largely restricted by topological domain boundaries, other types of loops are often formed between loci located far away from each other or even on different chromosomes, which makes their identification and subsequent analysis more difficult.

In many cases, these clusters are formed within specialized nuclear subcompartments enriched for specific factors. This spatial segregation within the nucleus allows for sharing the resources and for effective control of gene expression and silencing. However, it is still not known how this organization is achieved.

Further genome-wide studies on chromatin connectivity are needed to disentangle these complex networks and to properly understand mechanisms of their creation and their impact on genome functioning. In particular, single-cell techniques will be useful to identify networks present in a cell at a particular time point, and live-cell imaging may be able to elucidate the dynamics of these loops.

### Topological domains

Interphase chromosomes are partitioned into megabase-sized topologically associating domains (TADs), which take a very characteristic form of squares positioned along the contact map diagonal (Fig. 1d), indicative of high frequency of interactions between loci within a domain, and low frequency of contacts between loci located in different domains, even neighboring ones (Nora et al. 2012; Dixon et al. 2012). Even though TADs are believed to play an important functional role as they correlate with many chromatin-related features (such as epigenetic marks, gene expression, lamina associations, replication timing (Dixon et al. 2012), and regulatory domains (Symmons et al. 2014)), they only represent a summary of a population of cells, and it has recently been shown that in general, they are not present in individual cells. This apparent contradiction stems from the underlying chromatin dynamic: while TAD-like contact clusters exist in individual cells, they are in constant process of formation and reformation. In a sense, population TADs represent the general tendencies of these clusters and mechanisms that govern their dynamics, and thus, it is important to study their properties (see “Cell to cell variability” section for further details).

Similarly as in the case of loops, it is difficult to uniquely determine the number of domains and their size. Dixon et al. reported discovery of 2200 domains with a median size of 880 kb in mice (Dixon et al. 2012). Rao and colleagues analyzed eight different human cell lines and identified between 4000 and 9000 domains in each, with sizes ranging from 40 kb up to 3 Mb, and a median size of 185 kb (Rao et al. 2014), and ~ 3400 domains were identified in pooled single-cell Hi-C in mESC (Nagano et al. 2017).

An interesting characteristic of the domains is their boundary regions, which are enriched in a number of features, including CTCF (present in 76% of all boundaries), active transcription marks such as H3K4me3 and H3K36me3, nascent transcripts, repeat elements, housekeeping genes (present in ~ 34% of TAD boundaries), and transfer RNA (Rao et al. 2014; Dixon et al. 2012). Domain boundaries are closely linked to transcription. While they can be formed independently of transcription, it was recently shown that transcription is required for their proper maintenance (Hug et al. 2017) and that novel boundaries may emerge at the promoters of developmentally regulated genes (Bonev et al. 2017). It is currently not known whether it is the transcription (coupled with some additional factors) that allows the boundaries to be formed, or, conversely, the formation of domains (and other related structural units) enables gene expression. Alternatively, there might be some unknown mechanisms that underlie both of these processes.

Topological domains form a nested hierarchy spanning multiple scales, from short subTADs to metaTADs spanning large portions of chromosomes. Several approaches were developed to identify such higher-order TAD structures. In a recent study, a reciprocal insulation measure was used to identify this hierarchy. From the analysis of the distribution of domains at different levels of the hierarchy, the authors concluded that the scale of TADs does not differ from other scales, suggesting that from a structural point of view, TADs do not constitute a distinguished hierarchy level. Conversely, when functional features such as CTCF clustering, transcriptional coregulation during differentiation, or enrichment of active histone marks or promoter-enhancer contacts were analyzed, it was found that the TAD scale correlates well with these features. Taken together, these results suggest that the role TADs play in a genome is functional rather than structural (Zhan et al. 2017b).

### Compartments

The contact maps obtained in the original Hi-C study exhibited a very characteristic plaid-like pattern with large alternating blocks of enriched and depleted interaction frequencies (Fig. 1b, c) (Lieberman-Aiden et al. 2009). On this basis, the authors suggested that chromosomes are partitioned into two sets of DNA segments, such that loci from one set preferentially contact other loci from the same set (Fig. 1g). They also showed that these sets, termed compartments A and B, correspond to genomic features. Mainly, compartment A correlates with gene density, transcriptional activity, chromatin accessibility, and activating chromatin marks such as H3K36me3. These properties had led to the conclusion that the compartment A corresponds to open, accessible, and actively transcribed chromatin (Lieberman-Aiden et al. 2009). It was later shown that the localization of the B compartment is correlated with lamina-associated domains (LADs) and late replication timing, which suggests a proximity to the nuclear periphery (Ryba et al. 2010).

Rao et al. used high-resolution (25 kb) Hi-C data to discover that the compartments A and B can be further divided into subcompartments with distinctive features based solely on the chromatin interaction patterns. The compartment A comprises subcompartments A1 and A2, which are both gene dense, possess highly expressed genes and chromatin marks characteristic for active chromatin, such as H3K36me3, H3K79me2, H3K27ac, and H3K4me1, and have early replication times. The difference between subcompartments A1 and A2 is that the latter contains longer genes, has lower GC content, and finishes replication later than the former (in the middle of S phase, while A1 at the beginning of S phase). Similarly, compartment B can be partitioned into subcompartments B1 (with epigenetic marks indicative of facultative heterochromatin), B2 (containing mostly pericentromeric heterochromatin), B3 (enriched at nuclear lamina and depleted at the nucleolus-associated domains), and B4 (present solely on chromosome 19 and spanning only 11 Mb, containing many KRAB-ZNF superfamily genes) (Rao et al. 2014).

Initially, it was suggested that compartments form an architectural framework in which TADs operate by switching between active and inactive compartments as a method of gene regulation, for example during cell differentiation (Fraser et al. 2015a), but recent studies on cohesin suggest that compartments and TADs are formed by different, probably antagonistic mechanism (see “Cohesin” section).

### Chromosome territories

In some simple organisms, such as *Saccharomyces cerevisiae,* chromosomes are arranged loosely and they can highly intermix (Meaburn and Misteli 2007). In many others, for example in mammalian cells, they occupy separate territories in nuclei, called chromosome territories (CT, Fig. 1a, f) as observed by both microscopic and 3C methods (Cremer and Cremer 2010; Manuelidis 1990; Meaburn and Misteli 2007; Cremer et al. 2006; Lieberman-Aiden et al. 2009; Nagano et al. 2013). In these organisms, chromosomes exhibit preferential positions within the nuclei (Parada and Misteli 2002; Bolzer et al. 2005) and relative to each other (Parada and Misteli 2002; Boyle et al. 2001; Sengupta et al. 2008; Zhang et al. 2012). Generally, the position of CTs in the nuclei depends on cell type and is correlated with transcriptional activity (Misteli 2007; Lanctôt et al. 2007; Bickmore and van Steensel 2013), with the gene-rich chromosomes commonly tending to be located near the nuclear interior and gene-poor ones—near the nuclear periphery (Croft et al. 1999; Boyle et al. 2001).

Microscopic studies suggested a sponge-like model of CT architecture, where CTs comprise a number of chromosome domains (CDs) permeated by an interchromatin compartment (IC), with an intermediate layer of decondensed chromatin called perichromatin region (PR). In this model, IC forms a contiguous network of channels anchored at the nuclear pores and containing various factors related to transcription, splicing, DNA replication, and repair (Schermelleh et al. 2008; Markaki et al. 2010; Rouquette et al. 2009; Albiez et al. 2006; Cremer et al. 2006). This structure allows DNA contained within domains to access those factors, which is consistent with the fact that the aforementioned mechanisms take place at the CD surfaces (Cremer et al. 2012; Rouquette et al. 2010). A recent study showed that chromatin domain clusters are composed from shell-like layers with different chromatin compaction levels, with a condensed core and more relaxed outer layers. Similarly, PRs and IC are also characterized by different chromatin compaction classes (Schmid et al. 2017). Based on the chromatin compaction and on the functional role of these domains, this model is also expressed in terms of two entities: active and inactive nuclear compartments (ANC and INC), where ANC comprises IC and PR, is characterized by low chromatin compaction, and is highly enriched in activating epigenetic marks and RNA Pol II, whereas INC consists of condensed CDCs and is enriched in silent chromatin (Cremer et al. 2015). Interestingly, a recent study found that the chromatin fiber organization is uniform across regions of different compaction and takes a form of a disordered polymer of 5 to 24 nm in diameter. This suggests that the genomic domain condensation is a result of denser chromatin fiber packaging rather than different higher-order organization, such as 30 or 120 nm fibers that were posited in the past (Ou et al. 2017).

The contacts between CTs were thought to be restricted by IC, which only allowed for rare inter-chromosomal contacts (Cremer and Cremer 2001). In these interactions, a specific locus (e.g., a regulatory element) is located on a loop extending from the CT and reaching another locus on a different chromosome, as exemplified by the gene-rich major histocompatibility complex (Volpi et al. 2000) or the HoxB gene cluster (Chambeyron and Bickmore 2004). In both these cases, the looping is correlated with upregulation of the corresponding genes. In some instances, these inter-chromosomal contacts are correlated with chromatin decondensation which is not only required for increasing DNA accessibility, but also explains how these gene clusters can reach distant loci (Chambeyron and Bickmore 2004). However, this is not always the case. For example, it was found that in the HoxD cluster, some looped-out gene loci were still condensed, and, conversely, some decondensed loops were located within CTs (Morey et al. 2007).

Later studies suggested that the chromosomal intermingling is much more frequent than previously thought (Branco and Pombo 2006; Misteli 2010; Visser et al. 2000). As an example, Branco et al. devised a high-resolution in situ hybridization technique to capture the intermingling of CTs in human lymphocyte cells, showing that these frequent interactions are correlated with chromosome translocations. They also detected a presence of transcription machinery at the intermingling area and showed that transcription inhibition leads to a significant decrease of CT intermingling, demonstrating a strong link between inter-chromosomal contacts and gene expression (Branco and Pombo 2006). All these discoveries suggest that the CT intermingling is an intrinsic property of chromatin which plays an important role for genome stability and function (Branco and Pombo 2006; Gasser 2002; Kleckner et al. 2004). In concordance with these results, a recent study noted considerable interchromosomal mixing (5–10%) (Stevens et al. 2017).

In contrast to these results, the authors of a recent Hi-C study suggested that there are no inter-chromosomal regulatory contacts in mammalian immune cells and argued that functional interactions identified in earlier works might be due to technical and biological biases. This assertion is based mainly on the fact that large proportion (75–90%) of the *trans* contacts contain regions that were located within centromeres or telomeres, contained genomic repeats, or had mappability issues, and that when filtered out, the remaining data showed no contact enrichment between pairs of loci that were previously reported to be linked (Johanson et al. 2017). The results of this study stay in a stark contrast with numerous previous works, and a careful analysis and cross-validation should be performed to assess its findings. In any case, the study raises important questions about technical and methodological biases in 3C data and their interpretation, which were also made independently by others (see “3C vs. microscopy” section).

Initial single-cell Hi-C maps confirmed that chromosomes assume well-defined territories and suggested that active regions are located on a limited, relatively constant CT surface, where they are able to contact other regions both *cis* and *trans*. The contact networks varied significantly between cells. As in the case of intrachromosomal contacts, interchromosomal interactions preferentially took place between regions belonging to the same compartment. Interestingly, the number of chromosomes that a particular chromosome is interacting with was relatively unchanged and did not depend on the chromosome size (Nagano et al. 2013). A different view was suggested in a more recent study, where it was found that organization of compartments, LADs, and active genomic elements in mESC is consistent across cells and consists of three layers: compartment B regions on the surface, an inner A compartment ring, and, innermost, another compartment B layer around the nucleoli (Stevens et al. 2017). The spatial separation of compartments was additionally confirmed by microscopy (Wang et al. 2016b).

It is already well-known that gene positioning within this complex, multilayer structure plays an important role for its regulation, as was observed for example during differentiation (X. Q. D. Wang and Dostie 2017) and in disease (Meaburn et al. 2009). Genome-wide, there is a high correlation between gene expression level and its localization in respect to CT surface and its depth within compartment A (Stevens et al. 2017; Peric-Hupkes et al. 2010). Yet, the details of how a position of a gene in this multilayer structure impacts it regulation are not yet well-understood. In particular, while some studies have shown a link between gene repositioning and its silencing or activation, in others, no such link was found, suggesting a gene-specific behavior (Shachar and Misteli 2017). In one study, a high-throughput method for mapping gene positions within nucleus was developed, and it was found that replication is one of the main factors that drives gene repositioning (Shachar et al. 2015).

### Cell to cell variability

As indicated by FISH studies, there is a large variability between conformations that different cells exhibit even between cells of the same type, suggesting that genome organization is highly dynamic and variable (Rapkin et al. 2012; Schoenfelder et al. 2009). This was confirmed by the initial single-cell Hi-C experiment. Even though it suffered from the data scarcity (only ~ 1000 contacts per cell), it was enough to observe that the higher-order organization (and inter-domain contacts in particular) is highly variable between cells (Nagano et al. 2013). This calls the validity of drawing conclusions about genome organization in individual cells from the population-based data into question. Simply put, population-based data represent an average over thousands or millions of cells, and thus may lack important features present in individual cells.

A more recent study attempted to study the relation between domains in individual cells and in the population data (Flyamer et al. 2017) and discovered that while the TAD-like contact clusters were detectable in single-cell heatmaps they were highly variable, did not align with population TADs, and often crossed their boundaries. However, when pooled together, the contact clusters averaged into TADs. This means that TADs do not reflect structures present in individual cells, but rather represent population tendencies. As mentioned above, computer simulations based on the loop extrusion model also showed that variability in position and size of loops is necessary to reproduce the structural features observed in population Hi-C heatmaps (see “Cohesin” section).

These results were confirmed by computational modeling based on single-cell Hi-C, which has shown that the structure of loops and domains is very variable between cells (Stevens et al. 2017). For example, the conformations of a number of selected TADs were studied and it was found that a structure of a particular TAD in different cells may range from highly compacted to widely extended (Giorgetti et al. 2014; Stevens et al. 2017; Szabo et al. 2018). It was also discovered that this variability is related to transcriptional activity (Giorgetti et al. 2014; Zhan et al. 2017a). In the case of loops, in one study, it was found that, in overall, the ~ 2800 loops that were identified were present in ~ 60% of the cells and that 33% of the 88 longest loops were not present in any of the cells (Stevens et al. 2017). These results are in agreement with FISH, which showed that specific loops may be present only in a population subset (Rao et al. 2014), and may simply reflect the dynamic nature of the loop extrusion process, or the fact that promoter-enhancer loops are often formed only temporarily for the expression initiation, and thus they are not be present in a cell at all times (Ulianov et al. 2017).

It was suggested that this variability is driven by multiple factors, such as active/repressed chromatin state, chromatin interactions with nucleolus, nuclear lamina and other nuclear structures, DNA clustering near nuclear micro-compartments such as nuclear speckles or transcription factories, and, finally, the stochasticity of the loop extrusion (Ulianov et al. 2017).

This stochastic nature of genome organization is thought to play an important biological role. For example, alternative conformations that a particular region may take may allow for regulatory adaptation in response to various stimuli, but further studies (possibly combining FISH, single-cell 3C, and live-cell imaging) will be required to fully assess its source, importance, and biological consequences (Ulianov et al. 2017).

## Architectural factors

The intrinsic, multiscale organization described above requires an orchestrated work of multiple factors (Mourad and Cuvier 2016). While many such factors were identified or suggested, there seem to be a general consensus that CTCF and cohesin play major roles. An important role is also played by nuclear bodies (NB) such as nuclear speckles or nucleolus which work on different scales, from mediating clustering of coregulated genes to being involved in separation of genomic domains.

### CTCF

One of the most important players in the genome organization is CTCF. It is known to be involved in various functions in genome: it both mediates and blocks long-range interactions (Phillips and Corces 2009), regulates gene expression by enhancer blocking activity of vertebrate insulators (Bell et al. 1999; Hou et al. 2008) and by acting as a barrier dividing active and silent chromatin (Chung et al. 1993; Cuddapah et al. 2009; Narendra et al. 2015), demarcates the chromosomal domains (Kim et al. 2007; Xie et al. 2007), and is involved in chromatin loop formation. CTCF also seems to be involved in the formation of lamina-associated domains (Guelen et al. 2008; Handoko et al. 2011).

The number of CTCF binding sites varies for different cell lines, but many studies suggest that most mammalian cell lines have approximately 30,000 to 70,000 binding sites (Chen et al. 2012; Wang et al. 2012; Cuddapah et al. 2009), with approximately 5000 of them being ultraconserved between mammalian species and tissues (Schmidt et al. 2012). Between 30 and 60% of CTCF sites are cell-specific (Cuddapah et al. 2009; Barski et al. 2007; Kim et al. 2007; Chen et al. 2008), and approximately 15% of CTCF sites are located in TAD boundaries (Handoko et al. 2011). The remaining sites are believed to be involved in mediating short-range intra-TAD interactions (Lin et al. 2012). For example, CTCF bound at enhancers and promoters is thought to interact with each other and facilitate enhancer-promoter interactions (Weintraub et al. 2017).

A number of studies attempted to assess the importance of CTCF on genome organization by using CTCF degradation systems and yielded somewhat different results. One early study discovered that CTCF depletion had only a mild effect on TADs (Zuin et al. 2014), but it was later shown that this effect was probably related to incomplete CTCF loss (Nora et al. 2017). More recently, two independent studies used auxin-inducible degron techniques to deplete CTCF in mESC (Nora et al. 2017; Kubo et al. 2017). In the former, it was found that CTCF depletion leads to almost complete loss of insulation between neighboring TADs. Interestingly, it had no visible effect on compartmentalization of active and inactive chromatin. The chromatin loops persisted, but with reduced strength (Nora et al. 2017). In the second study, the weakening of loops and preservation of compartments was also observed, but the effect on the domains was two-fold: while the LAD-associated domains disappeared, other domains—contradictory to the results of the former study—remained relatively stable, with only a slight weakening (Kubo et al. 2017).

The disagreement between these results may be due to several factors. First, an incomplete CTCF degradation may be at fault. By using intermediate doses of auxin, Nora et al. showed that the CTCF loss needs to be almost complete to trigger substantial TAD disruption (Nora et al. 2017). Secondly, using binary TAD calls is problematic as it does not allow for proper boundary insulation quantification, which may lead to exclusion of weak boundaries from the analysis and/or to overstate the role of the strongest domains. The existence of CTCF binding within domains may suggest that there are other factors necessary for boundary formation. On the other hand, within-domain CTCF binding may represent weaker or more temporal subdomains of the hierarchical folding, or, alternatively, it may correspond to separate mechanisms, such as loop formation. Further studies will be required to assess the importance of CTCF in establishing and maintaining TADs, study these effects, and to draw more confident conclusions.

### Cohesin

Cohesin is a protein complex closely linked to CTCF and which has a significant impact on the genome organization (Ong and Corces 2014). Initially, it was best known for its role in regulating the separation of sister chromatids during cell division and DNA repair (McNairn and Gerton 2008; Nasmyth and Haering 2009; Peters et al. 2008), but later, it was discovered that it also plays a functional role in transcription regulation, where it is usually coupled with CTCF (Fig. 1h) (Handoko et al. 2011; Xiao et al. 2011; Wendt et al. 2008; Parelho et al. 2008; Rubio et al. 2008; Stedman et al. 2008; Galli et al. 2013; Xie et al. 2013; Zlatanova and Caiafa 2009). It was implicated to facilitate enhancer-promoter interactions (Merkenschlager and Nora 2016; Kagey et al. 2010), and its occupancy is correlated with transcription (Ocampo-Hafalla et al. 2016). Cohesin can also act independently of CTCF, as was shown for example in estrogen-regulated transcription (Schmidt et al. 2010). This is concordant with a recent study in which it was found that CTCF is much more dynamic than cohesin, with significantly lower residence (~ 1 vs. 22 min) and rebinding times (~ 1 vs. 33 min), suggesting that they do not form a stable complex (Hansen et al. 2017).

Cohesin distribution on DNA is very dynamic—it is loaded by Nipbl at specific sites and is subsequently translocated to other regions. For example, in yeast and *B. subtilis*, cohesin loaded onto DNA at centromeres is relocated to chromosomal arm sites (Davidson et al. 2016; Wang et al. 2017), whereas in human cells, it is translocated from the Nipbl sites to CTCF sites (Busslinger et al. 2017). Studies using single-molecule imaging have shown that cohesin can rapidly diffuse along the DNA, but its mobility can be restricted by nucleosomes, nucleosomes arrays, and DNA-bound proteins (Davidson et al. 2016; Stigler et al. 2016). In particular, CTCF seems to be a very effective blockade. It is not yet known whether this is purely due to its physical size (which may stop or substantially slow down cohesin passage), or if it creates a high-affinity site for cohesin (Haarhuis et al. 2017). While it was suggested that cohesin can move along DNA by passive diffusion (Stigler et al. 2016), its movement is also transcription-dependent, as it can be pushed by RNA polymerase (Ocampo-Hafalla et al. 2016). In any case, this movement usually continues until cohesin either encounters a CTCF bound to DNA or until cohesin-associated protein Wapl releases it from DNA (Tedeschi et al. 2013; Davidson et al. 2016; Busslinger et al. 2017).

An interesting and important role for cohesin was suggested in a loop extrusion model, in which cohesin-based complex is implicated to extrude chromatin loops by sliding on DNA until a properly oriented CTCF motif or another extruding complex is detected (Sanborn et al. 2015; Fudenberg et al. 2016; Barrington et al. 2017). While relatively simple, this mechanism is sufficient to produce intrinsic chromatin folding and create separated domains. It is not yet clear what is the driving force of the mechanism, what role cohesin exactly plays (whether it is a motor extruding the chromatin, or just a ring through which chromatin passes), and how many CTCF and cohesin units are involved in the extrusion. This model was validated both experimentally, by re-engineering loops and TADs in a predictable fashion using short targeted mutations (Sanborn et al. 2015), and computationally, by polymer physics simulations which showed that the obtained models are concordant with the experimental observations (Fudenberg et al. 2016; Naumova et al. 2013).

Initial studies have shown only limited effect of cohesin depletion on the genome organization (Zuin et al. 2014; Seitan et al. 2013; Sofueva et al. 2013). This might be due to incomplete cohesin loss (similarly as in the case of CTCF depletion studies) or low-resolution data used in these works, making it impossible to distinguish between domains related to loops and compartments (Rao et al. 2017). More recent studies showed more dramatic effects. In one of them, AID was used to degrade RAD21, a core component of cohesin complex, which prevented the cohesin from binding to DNA. It was discovered that while the distribution of CTCF and histone modification patterns remained unchanged, loop domains completely disappeared as a result, but were quickly restored after auxin withdrawal. Interestingly, the loops with enrichment of NIPBL binding, enhancers, promoters, and active histone marks (H3K36me3 and H4K16Ac) were restored significantly faster than loops depleted in these features, which also possessed repressive marks (H3K27me3 and H3K9me3). Interestingly, the compartmentalization become even stronger (Rao et al. 2017), consistent with a previous study in which chromatin compaction increased after CTCF and cohesin knock-down (Tark-Dame et al. 2014). In the second study, it was observed that the depletion of Scc1, a cohesin subunit, in zygote cells led to almost complete loss of loops and TADs, with an 1.8-fold compartmentalization increase (Gassler et al. 2017).

Two studies took slightly different approach and attempted to assess the effect of Nipbl loss. While the results of one of them (Schwarzer et al. 2017) were concordant with Rao and almost complete loss of TADs and loops was observed. Interestingly, there was a small number of persisted loops (61 out of ~ 1000). They were much longer (median length 23.15 vs 0.275 Mb), highly enriched with superenhancers, and entered *trans* contacts with each other. Interestingly, these changes did not have any impact on gene expression. The second study gave different results. Mainly, it was found that Nipbl depletion led to formation of shortened loops. This difference may be due to the fact that Nipbl-independent cohesin loading also occurs, which can mitigate the effect of Nipbl loss (Stigler et al. 2016; Davidson et al. 2016).

Given the importance of cohesin presence and proper loading, two groups examined the effect of Wapl depletion, which was previously shown to prolong cohesin residence time more than 10-fold (Tedeschi et al. 2013). Both studies noted that the loops were significantly longer and that the number of inter-domain contacts increased for neighboring domains, consistently with the loop extrusion model. Interestingly, these loops share the anchors with “normal” loops, which confirms CTCF as a blocking element (Haarhuis et al. 2017; Gassler et al. 2017). These results show that cyclic cohesin loading and unloading has an important role in genome organization, and disruption of either of these mechanisms leads to significant structural changes.

Taken together, these results suggest that chromatin loops are very dynamic and relatively short-lived entities which are continuously extruded and dissolved. This view is consistent with FISH (Sanborn et al. 2015; Williamson et al. 2014) and single-cell studies (see “Cell to cell variability” section). The loops observed in population-based data represent merely the final stages of loop enlargement.

The loop extrusion is an attractive model of loop formation that is able to explain the structural features observed in experimental data and to predict effect of various changes such as removal of CTCF binding site or changes in cohesin DNA residency time. In particular, the model is consistent with the dynamic nature of the chromatin loops, as models with static loops cannot reproduce experimentally observed structural features (Fudenberg et al. 2016; Sanborn et al. 2015; Naumova et al. 2013).

One of the crucial finding of these studies is that domains and compartments are emanations of two separate, antagonistic mechanisms—preferential association of epigenetically and transcriptionally similar regions in 3D resulting in compartmentalization, and local, cohesin dependent, 1D extrusion leading to chromatin loops and domains and resulting in a segregation of enhancers and promoters.

### Nuclear bodies

A role in genome organization is also played by nuclear bodies, membrane-less microenvironments that play various roles in genome (Dundr 2012; Mao et al. 2011). For example, around 4% of the mammalian genome is associated with nucleoli and form nucleolus-associated domains, which contain mostly transcriptionally inactive regions enriched with satellite repeats (Németh and Längst 2011).

In a recent study, it was found that nucleolus and nuclear speckles can act as interchromosomal hubs driving genome organization, as multi-way networks of interactions related to these bodies were detected. Interestingly, it was found that the active regions cluster around nuclear speckles, while inactive ones around nucleolus (Quinodoz et al. 2017; Brown et al. 2008). These associations reflect strong preferences, as there is a high correlation between transcriptional output of a gene and its distance to a nuclear speckle (Quinodoz et al. 2017), and ~ 4% of the mammalian genome is located in nucleoli-associated domains (NADs) that contain mostly transcriptionally inactive regions enriched with satellite repeats (Németh and Längst 2011).

Cajal bodies (CB), another type of NB, are preferentially located at the CT boundaries near the inter-chromosomal interfaces (Wang et al. 2016a; Gall 2000). They are also involved in clustering and upregulation of small nuclear and small nucleolar RNA (snRNAs, snoRNA) and highly expressed histone genes, drive their preferential positioning inside the nucleus and mediates chromosome 1 topology (Machyna et al. 2014; Wang et al. 2016a; Sawyer et al. 2016; Stanek and Neugebauer 2006). CBs are also involved in splicing, but they do not have a significant impact on global expression levels.

To conclude, nuclear bodies seem to be one of the main factors responsible for genome compartmentalization, specifically on the interchromosomal scale, by bringing together loci with similar transcriptional activity.

## Genome reorganization

Genome is not a fixed entity, and it undergoes various changes that add an additional layer of dynamic. With the exception of loop and domain modifications, which can be triggered by mutations or epigenetic factors and typically have only local impact, the processes described below occur in cells on a regular basis and are usually linked with global genome topology reorganization.

### Cell cycle

The loop- and TAD-based genome organization described above is not fixed, and it undergoes dramatic changes during mitosis, when the chromosomes become highly condensed and transcriptionally inactive. Different groups studied the changes occurring over transition from interphase to metaphase employing both population (Naumova et al. 2013) and single cell based experiments (Nagano et al. 2017).

The former study shows complete disappearance of compartments and TADs during metaphase and suggests that they are recreated in early G1 phase. Based on a comparison of Hi-C heatmap and polymer simulations, a two-step model of mitotic chromosome condensation was suggested. In this model, the consecutive loop anchors are first brought together to form compressed arrays, and then, they are axially condensed. Some things remain unknown—for example, it is not known whether there is some scaffolding or chromosomal axis, similar to the one observed, e.g., in lampbrush chromosomes.

The latter study used thousands of single-cell Hi-C (Nagano et al. 2017; Nagano et al. 2015a) contact maps to study the continuum of chromosomal conformations representing the whole cell cycle. It shows that while the domain boundary locations are generally unchanged between G1, S, and G2, their insulation strength varies dynamically. The maximum is reached during G1, then starts to decrease when replication begins, plateau at mid-S, and stays unchanged until mitosis, when it disappears again. Interestingly, the dynamic of compartmentalization is different: it is weak in G1, and then, it starts to steadily increase in S and G2, with a drastic drop before the mitosis. This difference constitutes another confirmation that the domains and compartments are formed through different mechanisms. It was also discovered that the re-formation of compartments and domains is correlated with DNA replication timing, and that the chromatin loops are generally stable during the interphase. However, it is still unknown precisely what biophysical mechanisms stand behind the chromatin reorganization through the cell cycle.

### Embrional development

Sperm cells are interesting models to study as they are significantly smaller than typical cells, and thus require much higher level of chromatin condensation (which is mediated mainly by protamines and not histones). Interestingly, it was shown that sperm genome organization shares many features with other cell lines, such as mESC. Mainly, it contains a similar number (~ 23,000) of CTCF and cohesin bound sites, and the patterns of TADs and compartments it exhibits mostly overlap with the ones observed in mESC and fibroblasts (Jung et al. 2017; Battulin et al. 2015).

Recently, it was shown that the chromatin exists in a relaxed state after fertilization and architectural features such as TADs, loops, and compartments appear gradually during embryonic development, becoming stronger with the progressing development (Du et al. 2017; Ke et al. 2017; Flyamer et al. 2017; Hug et al. 2017).

Recent low-input Hi-C studies found that the TAD and compartment-related signal is absent in oocytes, start to emerge in zygotes and 2-cell stage embryos, become gradually more apparent in later stages, and is fully visible only around in 8-cell embryos (Ke et al. 2017; Du et al. 2017). Interestingly, it was also found that paternal and maternal chromosomes are spatially separated from each other also until 8-cell stage (Du et al. 2017). Another study tried to assess the relation between transcription and genome organization by comparing in situ Hi-C maps before and after zygote genome activation (ZGA). It was found that the structural features were mostly absent before ZGA, with the exception of ~ 180 regions that resembled TAD boundaries and were enriched for housekeeping genes. The activation of gene expression was followed by rapid TAD formation; it was, however, shown that while these two processes are closely linked, they are independent of each other (Hug et al. 2017).

In contrast to these results, a single-nucleus Hi-C study found the presence of chromatin loops and contact clusters in both oocytes and zygotic cells. While the identified contact clusters were highly variable and often crossed the population TAD boundaries, when pooled together, they averaged to population TADs. Interestingly, the compartments were absent in the maternal zygote, but were present in paternal zygotic cells and in oocytes, suggesting that their formation is independent of TADs (Flyamer et al. 2017).

The discrepancy between the above results was resolved by a later study (Gassler et al. 2017) in which bulk Hi-C datasets were re-analyzed using an aggregated analysis, where the contact maps are averaged over a set of pre-specified loci. In this case, a set of loops identified in a previous study in CH12-LX cells (Rao et al. 2014) and de novo TAD calls from more than 15 datasets and multiple cell lines was used. In all these cases, the presence of loops and TADs could be observed as early as one-cell embryo. The strength of these structures was shown to gradually grow, consistent with the original study results (Du et al. 2017; Ke et al. 2017).

### Cell differentiation

The structure of TADs undergoes only slight changes during cell differentiation. Approximately 70–80% of TAD boundaries are conserved during the mESC lineage transmission to intermediate NPC and to postmitotic neurons (Fraser et al. 2015a). It seems that a more important mechanism of cell differentiation is the switching of compartments A and B by TADs, to which approximately 36% of the genome is susceptible in at least one of 4 lineages considered in the study (mesendoderm, mesenchymal, neural progenitor, and trophoblast-like cells). This change is connected with a variation in the expression rates, with TADs switching to compartment A being upregulated, and those switching to compartment B downregulated (Dixon et al. 2015).

This domain reshuffling (as the compartment switching domain will probably change its position relative to other domains) may explain a well-studied fact that during the cell development, genes change their nuclear position (Peric-Hupkes et al. 2010; Schneider and Grosschedl 2007). Generally, gene loci change position from nuclear lamina to nuclear interior which allows to establish new intra- and inter-domain interactions associated with the lineage-specific transcription patterns, as it was demonstrated for Igh (Kosak et al. 2002) and Ebf1 (Lin et al. 2012) loci. This process was shown not only for individual loci, but also for multigene regions.

Another study showed that domains and compartments increased in size due to disappearance of some of their boundaries. This change was accompanied by changes in the compartmentalization patterns between NPCs and CNs, with a decreased number of interactions between compartment A domains, and increased contacts between B domains (Bonev et al. 2017).

An extensive reorganization also happens within TADs, which allows for precise lineage-specific transcriptional changes. This reorganization is carried out by two main mechanisms: rewiring of the promoter interactions and modification of chromatin states of the corresponding sites, which work concomitantly and are particularly strongly associated with cell-type-specific promoter interactions. Both these mechanisms are linked to changes in the gene expression, suggesting that they both play important regulatory function (Freire-Pritchett et al. 2017). In one study, it was found that less than half of the enhancers is shared between mESC and FLC. Out of these, only a fraction contacts the same promoters, and there is a drastic difference between sets of highly active enhancers in both cell types (Schoenfelder et al., 2015a). These results were confirmed by a more general study which analyzed interactomes of 17 human primary blood cell types, discovering that they are highly cell-type-specific and form a hierarchy closely matching the hematopoietic lineage, suggesting that related cell lines exhibit similar interaction networks (Javierre et al. 2016).

Another observed mechanism, independent of the transcription, is the decrease of polycomb-mediated contacts, as studied in the transition from mESC to neuronal cells. While very strong in mESC polycomb interactions become gradually weaker with the progressing differentiation, with the exception of a small set of genes, for which they maintain their strength or are even enhanced (Bonev et al. 2017).

To conclude, a major reorganization takes place during cell differentiation. On a coarse scale, domains switch compartments and change their preferential associations with other domains. This clustering of TADs can be viewed as a nested hierarchy of ‘metaTADs’ at higher organizational levels, and thus, the compartment switching can be represented as rearrangements of the metaTADs tree-like structure, which may be a helpful tool to study differences between various lineages (Fraser et al. 2015a). On a finer scale, an extensive rewiring of promoter contacts and chromatin state modifications allow for a more precise transcriptional control and activating or repressing lineage-specific genes.

### Chromosome X inactivation

One of the most prominent global reorganization events was identified in mammalian cells during chromosome X inactivation (XCI) (Rao et al. 2014; Deng et al. 2015). Recently, it was shown that the XCI is initiated by upregulation of non-coding Xist RNA, which induces chromosome silencing by recruitment to nuclear lamina (Giorgetti et al. 2016; Minajigi et al. 2015; Chen et al. 2016). This leads to loss of compartmentalization and TAD structure, and to formation of two superdomains separated by a macrosatellite repeat element DXZ4, as shown by 3C (Darrow et al. 2016; Chadwick 2008) and microscopy (Wang et al. 2016b) approaches. DXZ4 is located in the heterochromatin, but in response to the chromosome X inactivation, it is organized into euchromatin bound by CTCF (Horakova et al. 2012). Earlier studies suggested that this CTCF is involved in creation of large chromatin loops which keep the structure condensed and inaccessible to the transcription machinery, such as a prominent 13-Mb-long CTCF-mediated chromatin loop between DXZ4 and FIRRE loci and a 23-Mb-long interaction between FIRRE and G6PD (Rao et al. 2014; Tang et al. 2015), or related superloops spanning more than 70 megabases (Darrow et al. 2016; Rao et al. 2014). The formation of DXZ4 boundary is initiated by Xist, and its deletion results in the merging of the superdomains together (Darrow et al. 2016; Giorgetti et al. 2016). Interestingly, Xi silencing is not complete, with a number of escape genes cluster retaining DNA-accessibility and TAD-like structure (Giorgetti et al. 2016).

### Loops/domain modifications

**Fig. 2:**
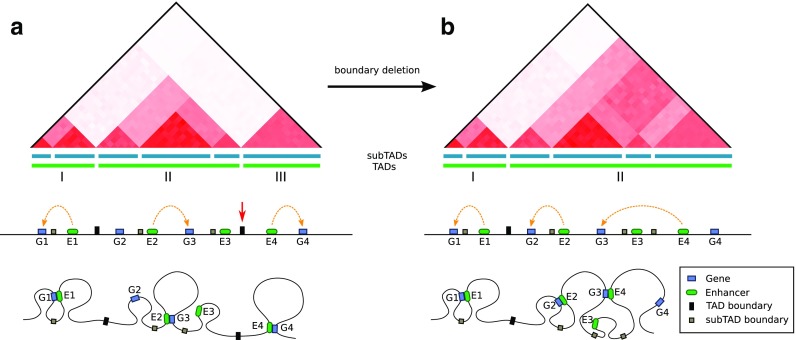
A toy example of a genome reorganization after a TAD boundary disruption, shown using 3 different perspectives: contact maps, genomic diagram, and a chromatin looping model (top, central and bottom row, respectively). **a** A sample region with three domains (marked with green bars and labeled I, II, and III) separated by TAD boundary elements (black rectangles) is presented. The domains are further divided into sub-domains (blue bars) separated by subTAD boundary elements (gray rectangles). Interactions between genes and enhancers are restricted to domains (E1-G1, E2-G3, E4-G4), but they can bypass the subdomain boundaries (E1-G1). b After the boundary disruption (marked with red arrow), former domains II and III merge together allowing for contacts between previously separated loci, as indicated by increased interaction frequency between the domains observed in the heatmap. Without the insulating barrier, enhancer E4 changes its target from G4 to G3, which disrupts prior interactions. In this example, G4 loses its enhancer while E2 gains a new target gene.

On a finer scale, it is known that mutations involving CTCF binding sites may disrupt the chromatin looping and domain structure which may influence the associated gene expression (Fig. 2) (Dowen et al. 2014; Guo et al. 2015b). In a recent study, Lupiáñez et al. (2015) demonstrated that structural variants, such as deletions and duplications disrupting the CTCF binding sites located at the TAD boundaries may lead to novel enhancer-promoter interactions and misexpression, resulting in pathogenic phenotypes. Interestingly, structural variants that did not overlap with the CTCF sites did not alter the genome organization at the region and did not influence the phenotype (Lupiáñez et al. 2015).

It was also shown that mutations disrupting domain boundaries are present in many types of cancer. In one study, it was found that recurrent microdeletions perturbing the domain boundaries in T cell acute lymphoblastic leukemia cells were sufficient to activate proto-oncogenesis (Hnisz et al. 2016).

Chromatin topology is very sensitive, and even a single SNP located within a CTCF binding site can lead to its alteration. Sanborn et al. used CRISPR/Cas9-based genome editing to show that even a single point mutation can lead to disruption of loops and domains (Sanborn et al. 2015). Tang and colleagues studied naturally occurring SNPs localized at the CTCF sites and also showed the domain disruption (Tang et al. 2015).

Moreover, there are also epigenetic mechanisms responsible for TAD boundary disruption. Flavahan et al. demonstrated that a mutation in a IDH gene may lead to hypermethylation of the CTCF and cohesin binding sites located at the domain boundary, which prohibits CTCF and cohesin recruitment and leads to loss of insulation between the domains. This allows a constitutive enhancer to interact with a prominent glioma oncogene, the receptor tyrosine kinase gene PDGFRA, located in a neighboring domain (Hnisz et al. 2016; Flavahan et al. 2016).

All these results suggest that disruption of chromatin loops and—most prominently—domains may lead to new inter-domain looping, which may lead to misexpression of important genes and have serious phenotypic consequences.

## Experimental methods

The level of technological advancement is one of the major factors restricting our ability to elucidate the inner workings of the genome organization. Implicit and explicit assumptions about tools we use may bias conclusions we draw from the data if not properly interpreted. While it is out of scope of this work to discuss the limitations and strengths of various techniques, below we shortly discuss the interplay between population- and single-cell based 3C methods and microscopy. We also discuss two recently developed techniques that took non-traditional approach for studying the chromosomal contacts.

### Single-cell approaches

Recent boom of single-cell 3C techniques brought many new insights and helped to clarify some old misconceptions. There are several advantages of these techniques. First, it allows to take a snapshot of an individual cell, which allows to identify which structural features are present in a specific cell at a particular time. Secondly, by comparing datasets from different cells, it allows to detect cell-to-cell variability, which is helpful for example in assessing which structural features are omnipresent in the cells, which in turn may suggest their crucial role. Thirdly, it also allows to study rare cells such as stem cells, oocytes or embryos, for which the amount of available material is limited. Lastly, single-cell approaches can be used to separate a mixed cell sample by karyotype and cell cycle state (Ramani et al. 2017).

Still, population-based approaches posses certain advantages. By analyzing numerous cells, they are able to detect rare events, which might be missed when only a limited number of cells are analyzed (Fudenberg and Imakaev 2017). While proper statistical methods to discern between biological and/or technical noise and true signal are required, even speculative results may give rise to biological hypotheses that could be later verified with more precise techniques. Moreover, these methods typically result in larger datasets, and thus, they are useful for whole-genome analyses.

### 3C vs. microscopy

One problem with the interpretation of the experimental data is that interaction frequencies obtained from 3C are not directly relatable to spatial distances measured by microscopy techniques such as FISH. Simply, FISH enables capture of the physical distance between a small number of selected loci in a single cell (and to approximate the distribution of this distance in a population if multiple cells are used). In turn, 3C techniques use crosslinking and proximity-based ligation to merge DNA fragments that were in spatial vicinity. For two fragments to be ligated, they need to be sufficiently close to each other. This capture distance depends on multiple factors, such as restriction frequency and efficiency. Thus, 3C measures the frequency with which a pair of loci is located closer than the capture distance. While interaction frequencies from 3C are often used as a proxy of spatial distance, these two quantities substantially differ, and using them interchangeably may lead to incorrect conclusions (Giorgetti and Heard 2016; Fudenberg and Imakaev 2017).

Another problematic issue is proximity ligation, one of the crucial steps in 3C techniques. It is performed after cross-linking and DNA cleavage by restriction enzymes, when the cross-linked molecules preferentially ligate with each other in solution. It was shown, however, that in the standard 3C protocol, a significant part of nucleus remains intact and constrains the extraction of DNA. This means that 3C signal represents mostly contacts reflecting the higher-order DNA organization in non-lysed and/or not drastically disrupted nuclei rather than complexes of isolated molecules, as expected (Gavrilov et al. 2013). On the other hand, it was shown that a significant portion of interchromosomal contacts in 3C experiments is product of spurious in-solution ligation, which introduces noise and decrease reproducibility between replicates (Nagano et al. 2015b).

Aside of experimental issues, there are also more systemic issues. For example, the reliance of 3C on the capture distance means that 3C-based techniques will not be able to capture longer contacts, e.g., those formed around nuclear bodies (Quinodoz et al. 2017). Thus, for example, 3C may not be able to detect strong clusters centered around these bodies and a seeming discrepancy between results obtained from other techniques may occur. All these considerations suggest that we should carefully think about what the 3D data actually measures, how to properly interpret data from different techniques, and how to design cross validation studies.

### Ligation-free methods

A helpful input in this regard can be provided by recently developed, alternative approaches that do not rely on proximity ligation to produce genome-wide data on chromatin contacts.

One of such method is genome architecture mapping (GAM), a method that extracts thin nuclear sections from individual cells using cryosectioning and laser microdissection, and create corresponding genomic profiles by sequencing their DNA content (Beagrie et al. 2017). The authors applied this technique to mESC and found that it is able to reproduce structural features known from 3C methods. In particular, an extensive network of 4.5 million interactions involving active genes and an enrichment of contacts between enhancers and promoters were observed. One of the major strengths of the method is its capability to detect multiway interactions. To this end, the authors identified and studied ~ 100,000 of the strongest triplet interactions. It was found that these interactions span large distances, with ~ 80% of them spanning more than 30 Mb. These triplets were significantly enriched for super-enhancers or combinations of super-enhancer and highly transcribed TADs. Interestingly, there was no enrichment for normal enhancers, suggesting that typical enhancer is involved in simpler, pairwise interactions, whereas super-enhancers interact with multiple sites simultaneously (Beagrie et al. 2017). While the method possesses many advantages over 3C methods, it also has some drawbacks—it may be susceptible to biases related to different nuclear shapes of the cells analyzed, and it may be time-consuming to extract sections from individual nuclei (Finn and Misteli 2017).

Another example of such techniques is Split-Pool Recognition of Interactions by Tag Extension (SPRITE) (Quinodoz et al. 2017). In this technique, cells are crosslinked and the chromatin in individual isolated nuclei is fragmented. Then, a series of split-pool tagging rounds is performed. In every round, the material is splitted between a number of wells and the molecules within every well are uniquely tagged, and subsequently pooled together. As molecules from a crosslinked complex will always split to the same well, they will have identical set of tags (or barcode), whereas molecules from different complexes, while may be able to share some of the tags due to random assignment to the same well, in general will posses different barcodes. Finally, the contacts are identified by sequencing molecules with identical barcodes. SPRITE showed that transcriptionally active DNA cluster around nuclear speckles, whereas the inactive and centromeric regions organize around the nucleolus. This method also allows to detect long range (> 100 Mb) and interchromosomal interactions, such as interactions between loci belonging to compartment A (thousands of interactions), gene clusters (> 75 contacts), and simultaneous interactions between consecutive loops (several) (Quinodoz et al. 2017).

Both these methods are able to closely reproduce features known from both 3C techniques (they exhibit very similar contact maps as Hi-C, compartments, chromatin loops) and microscopy (e.g., the role of nuclear bodies on the genome organization) providing independent verification of the existence and importance of these features and the corresponding structures. Moreover, they are particularly useful for detecting multi-way interactions and other features of genome organization, such as association with nuclear bodies, and may be helpful to reconcile 3C and microscopy techniques.

## Conclusions

For a long time, genome was treated mostly as a one-dimensional sequence of DNA, but in order to understand how it functions, it should be regarded as a complex, multi-level, three-dimensional system. Recently, a lot of effort was put to further investigate the inner workings of the DNA organization. While these studies brought out many new insights about processes and mechanisms involved in the genome functioning, there are still many unanswered questions.

Many of the discoveries described above were made using population-based methods, in which gathered data represent an average over millions of cells. Recent explosion and further improvement of single-cell techniques will allow to assess the structural dynamics of the genome topology during the cell cycle, and to better understand the genome organization variability across the cells. Technological developments in high-resolution and high-throughput microscopy made it possible to track the regions-of-interest motion in vivo*,* enabling us to precisely observe the small-scale genome topology readjustments in response to various stimuli. Theoretical models and considerations such as polymer physics simulations played an important role in verification of the biological hypotheses, and also helped to formulate new models that can explain the phenomena observed in experiments.

Currently, we possess a wide arsenal of experimental and computational techniques, and while the new ones are developed, the existing ones are continuously perfected. It is now important to properly assess their strengths, but also their technical and biological limitations. Integration of all these types different of data will lead to new insights providing a better understanding of the genome as a whole. In perspective, they may also contribute to the development of new diagnostic tools and therapies.
